# Bumetanide attenuates sevoflurane‐induced neuroapoptosis in the developing dentate gyrus and impaired behavior in the contextual fear discrimination learning test

**DOI:** 10.1002/brb3.2768

**Published:** 2022-10-02

**Authors:** Kai Wei, Yiheng Liu, Xiamin Yang, Jin Liu, Yuan Li, Meng Deng, Yingwei Wang

**Affiliations:** ^1^ Department of Anesthesiology, Huashan Hospital Fudan University Shanghai China; ^2^ Happy Life Tech Shanghai China

**Keywords:** apoptosis, bumetanide, dentate gyrus, sevoflurane

## Abstract

**Introduction:**

Sevoflurane acts as a gamma‐aminobutyric acid subtype A receptor agonist and can induce widespread apoptosis of immature dentate granule cells in postnatal day 21 mice. The dentate granule cells of postnatal day 21 mice undergo a developmental stage when gamma‐aminobutyric acid (GABA) shifts from inducing the depolarization of neurons to causing hyperpolarization. However, it is unclear whether sevoflurane induces apoptosis of immature granule cells by facilitating the depolarization or hyperpolarization of neurons.

**Methods:**

We utilized bumetanide, an Na^+^–K^+^–2Cl^−^ cotransporter isoform 1 (NKCC1) antagonist, to determine whether the NKCC1‐mediated GABA depolarization of neurons plays a role in sevoflurane‐induced neuroapoptosis. We also investigated whether sevoflurane exposure is related to long‐term cognitive dysfunction in postnatal day 21 mice and explored the possible protective effects of bumetanide.

**Results:**

Bumetanide attenuated the sevoflurane‐induced apoptosis of dentate granule cells in postnatal day 21 mice. Exposure to sevoflurane at postnatal day 21 mice did not affect their motor ability or anxiety level, and it had no effect on spatial learning or memory functions. However, sevoflurane exposure at postnatal day 21 impaired the pattern separation ability in the contextual fear discrimination test; bumetanide mitigated this effect of sevoflurane as well.

**Conclusion:**

Bumetanide attenuates sevoflurane‐induced apoptosis and is a promising prospect for protecting against anesthesia‐induced neurotoxicity in the developing brain.

## Graphical abstract

Bumetanide attenuates sevoflurane‐induced apoptosis of granule cells in the dentate gyrus of postnatal day 21 mice. Exposure of mice to sevoflurane at postnatal day 21 does not affect their motor ability or anxiety level, and it has no effect on spatial learning or memory functions. Bumetanide alleviates sevoflurane#x02010;induced performance impairment in the contextual fear discrimination learning test.

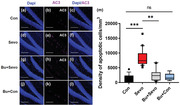

## 1 INTRODUCTION

It is estimated that millions of infants and young children receive surgery or imaging examinations under general anesthesia every year (Walkden et al., 2020). However, because nearly all commonly used general anesthetics can induce widespread neuronal apoptosis among different species, the safety of general anesthetics has attracted much attention (Brambrink et al., 2010, 2012; Deng et al., 2014). Despite numerous studies, the mechanisms involved in anesthesia‐induced neuroapoptosis remain to be elucidated.

The inhaled anesthetic sevoflurane acts mainly as an agonist of gamma‐aminobutyric acid (GABA) receptors and induces most of its effects through activation of the gamma‐aminobutyric acid subtype A (GABA_A_) receptor (Garcia et al., 2010; Rudolph & Antkowiak, 2004). Activation of the GABA_A_ receptor allows Cl^−^ ions to flow across cell membranes (Kaila et al., 2014; Khirug et al., 2008). In the mature central nervous system, GABA‐activated Cl^−^ channels evoke hyperpolarizing and inhibitory responses due to the high expression of K^+^–Cl^−^ cotransporter isoform 2, a neuron‐specific chloride extruder (Rivera et al., 1999). However, immature neurons prominently express Na^+^–K^+^–2Cl^−^ cotransporter isoform 1 (NKCC1); as a result, the intracellular Cl^−^ concentration is increased. When the GABA_A_ receptor is activated, an efflux of Cl^−^ leads to neuronal depolarization (Yamada et al., 2004). Our previous study identified that the vulnerability to anesthesia‐induced neuroapoptosis depended on the age of the neurons rather than the age of the animal, and immature neurons in the dentate gyrus (DG) were especially susceptible during the first 1–2 weeks of cellular life (Hofacer et al., 2013). At 1–2 weeks, granule cells in the DG undergo a stage when the effect of GABA shifts from depolarizing to hyperpolarizing the neuronal membrane (Toni & Schinder, 2015). However, it is unclear, whether sevoflurane modifies the dentate granule cells at this particular age by facilitating the depolarization or hyperpolarization of neurons.

In this study, we utilized the NKCC1‐specific antagonist bumetanide to determine whether NKCC1‐mediated GABA depolarization of the neuronal membrane plays an essential role in sevoflurane‐induced neuronal apoptosis. We hypothesized that bumetanide could alleviate sevoflurane‐induced neuroapoptosis in the developing DG. In addition, because the hippocampal DG is strongly associated with learning and memory functions, and immature granule cells in the DG at postnatal day 21 are most susceptible to neuroapoptosis induced by anesthetic (Hofacer et al., 2013), we investigated whether sevoflurane exposure is related to long‐term cognitive dysfunction in postnatal day 21 mice and explored the possible protective effect of bumetanide.

## 2 MATERIALS AND METHODS

### 2.1 Animals

All animal procedures were approved by the Institutional Animal Care and Use Committee of the Shanghai Medical College at Fudan University. All mice experiments were conducted in accordance with the Institutional Guidelines and Regulations of Fudan University. Pregnant C57BL/6J mice (embryonic day 18) were bought from the Center of Experimental Animals, at Fudan University. Newborn mice were weaned at postnatal day 21 and isolated from their mother. Mice were housed in a standard environment with free access to food, in a 12:12h day/night cycle at 22 ± 2°C.

### 2.2 Anesthesia

Postnatal day 21 C57BL/6J mice were divided randomly into control (Con), sevoflurane (Sevo), Bumetanide + Sevoflurane (Bu + Sevo,), Bumetanide + Control (Bu + Con) groups, and Bicuculline + Sevo group. The anesthesia procedure was conducted as previously described (Hofacer et al., 2013). Mice in the Sevo group were subjected to 3% sevoflurane in a mixture of 30% oxygen and 70% nitrogen for 6 h in an anesthetic box. Mice in the Con group were exposed to 30% oxygen and 70% nitrogen for 6 h in the same chamber. Mice in the Bu + Sevo group were injected intraperitoneally with 5 μg/kg of bumetanide 30 min before exposure to sevoflurane. Mice in the Bu + Con group were administered 5 μg/kg of bumetanide only and fasted in room air for 6 h. Mice in Bicuculline + Sevo group received 1 mg/kg bicuculline intraperitoneally 30 min before sevoflurane exposure.

### 2.3 Histology

Upon the completion of treatment, the mice were euthanized with sevoflurane and transcardially perfused with 0.9% NaCl, followed by overnight fixation using 4% paraformaldehyde in phosphate‐buffered saline (PBS). After fixation, the mice brains were treated using 20% and 30% sucrose–PBS solutions. Sagittal slices (40 μm) containing the hippocampal DG were prepared as previously described (Wei et al., 2019). For activated cleaved caspase‐3 (AC3) immunostaining, the brain slices were immersed in quick antigen retrieval solution for frozen sections (Boster; Wuhan, China) at 100°C for 5 min. The sections were then treated with 2 M HCl for 20 min at room temperature and washed in a 0.3 mM phosphate buffer. Subsequently, the slices were treated with blocking buffer for immunological staining (Boster; Wuhan, China) for 2 h and stained with rabbit anti‐AC3 antibody (9661; Cell Signaling Technology, Danvers, USA) overnight at 4°C. After three rinses with PBS, the sections were incubated with Alexa Fluor 647 goat anti‐rabbit secondary antibody for 2 h at room temperature. DAPI (4,6‐diamino‐2‐phenyl indole) was used to counterstain the nucleus. The stained sections were visualized using a Leica TCS SP8 confocal laser scanning microscope. The numbers of AC3 were traced at 20× magnification and quantified using the optical dissector method, as described previously (Howell et al., 2002).

### 2.4 Open‐field test

The open‐field tests were conducted at P49 in accordance with published protocols (Kraeuter et al., 2019). The mice were placed in the testing room 1 h before the experiment to allow them to habituate to the environment. The mice were then placed into the central zone of the box and allowed to explore the test area for 15 min. A video tracking program was used to record and analyze the total distance traveled, time in the center area, and the percent distance traveled in the center of the area.

### 2.5 Morris water maze

The Morris water maze (MWM) test was performed 1 day after the open‐field test was finished. The apparatus is a white water pool of 120 cm diameter and 50 cm height. Four different starting points were labeled on the outside of the pool in the target (T), left (L), opposite (O), and right (R) quadrants. A submerged platform was placed 1 cm below the water in one of the quadrants. Distinct visual cues were located around the pool and maintained unchanged throughout the test procedure. Each group of mice received four training trials per day for 5 consecutive days. In each trial, the mice were placed gently into the water at one of the four starting points, with their heads facing toward the wall, and allowed 60 s to locate and climb onto the platform. The time it took the mice to identify the platform was defined as the escape latency. If the mice failed to find the platform within 60 s, they were guided onto the platform and allowed to remain on it for 15 s. On the sixth day (test day), the platform was removed. The mice were placed randomly into the water and allowed to swim for 60 s. The number of times that mice swam across the target quadrant and the amount of time that the mice stayed in the target quadrant were measured using a video tracking system.

### 2.6 One‐trial contextual fear conditioning and contextual fear discrimination learning test

The one‐trial contextual fear conditioning test was performed 5 days after the MWM. The protocol was adapted from a previous report (Germer et al., 2019). Briefly, the mice were individually placed into a transparent Plexiglas cage with a stainless‐steel wire floor. For the context‐A test (Box A), the lights in the box were on, metal floor was exposed, and a mild lemon scent was utilized as an olfactory cue. After habituation for 185 s, the mice received an electronic foot shock (2 s, 0.8 mA) through the floor grid. Fifteen seconds after the shock, the mice were returned to their cages. The floor grid and cage walls were cleaned using 75% ethanol between trials. Twenty‐four hours after the context‐A test, the mice were again placed into the context‐A box and allowed to explore for 180 s without electronic shock (Box B). On the third day, the mice were placed into the context‐C box (Box‐C). The light in the context‐C box was off, two walls of the cage were covered by plastic panels, and the stainless‐steel grid floor was covered with cardboard. No electronic shock was delivered in the context‐C test.

To test the ability of the mice to discriminate between similar contexts, the contextual fear discrimination learning test was performed following a one‐trial contextual conditioning. The protocols were performed according to a previous study (Scobie et al., 2009) with slight modification. Both the context‐B and context‐A tests had an exposed stainless‐steel floor. However, the context‐B test had the walls of chamber covered with plastic panels, the light was turned off, and the chamber door was ajar during the test. The olfactory cue in context B was a mild pineapple scent, and a nonalcoholic antiseptic was used to clean the chamber between trials. On the fourth day of the experiment, the mice in each of the three groups were placed into the context‐A box for 180 s, and a shock was administered. From the fifth day, mice were subjected to the conditioning context (context A) with an electronic shock. After 1 h, the mice were put into the similar context (context B), in which they were allowed to explore for 180 s but did not receive a shock. The freezing behaviors of the mice in context A and context B were used as the assessment of discrimination until the difference was significant among the Con, Sevo, and Bu + Sevo groups.

### 2.7 Statistical analyses

All data were analyzed using SPSS 21.0 software (IBM). Normality was tested with the Shapiro–Wilk test. For parametric data, statistical results were assessed by an unpaired *t* test or analysis of variance (ANOVA) with Fisher's post hoc test, where appropriate. For nonparametric data, data were compared using the Kruskal–Wallis test followed by post hoc analysis with the Bonferroni correction between multiple groups and the Mann–Whitney *U* test between two groups. *p* < .05 was considered statistically significant.

## 3 RESULTS

### 3.1 Bumetanide attenuates sevoflurane‐induced granule cell apoptosis in the DG of postnatal day 21 mice

**FIGURE 1:**
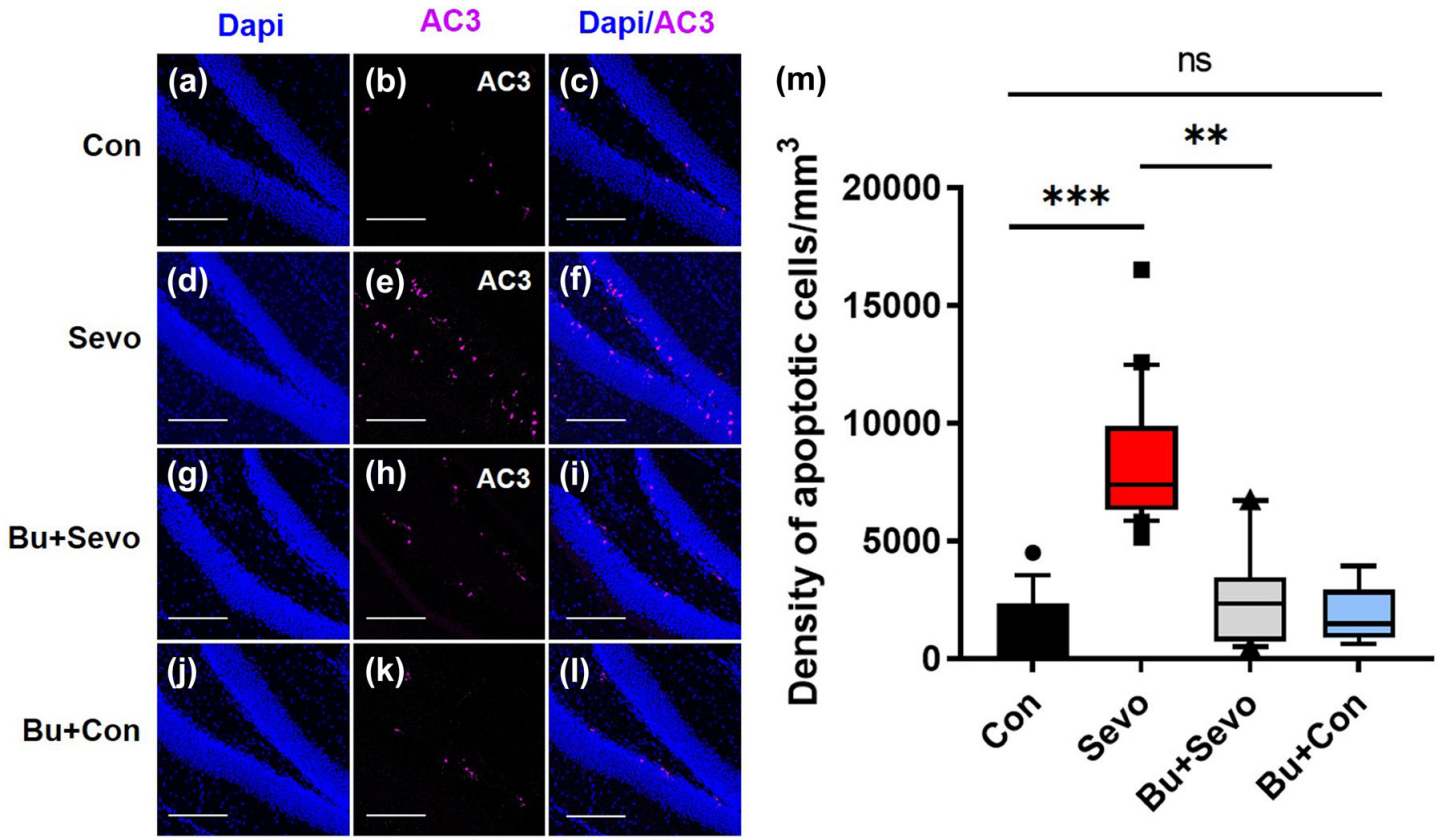
Bumetanide attenuates the induction of apoptotic granule cells by sevoflurane in the dentate gyrus (DG) of postnatal day 21 mice, as shown by immunostaining for AC3 and the nucleus in the Con (a)–(c), Sevo (d)–(f), Bu + Sevo (g)–(i), and Bu + Con (j)–(l) groups. Scale bars = 100 μm. (m) Box plot showing the density of apoptotic cells in the Con (*n* = 9), Sevo (*n* = 10), Bu + Sevo (*n* = 11), and Bu + Con (*n* = 8) groups. Boxes are 25th−75th percentiles, whiskers are 10th−90th percentiles, and closed circles depict outliers. ^***^ *p* < .001, ^**^ *p* < .01 versus Sevo group, Kruskal–Wallis test followed by post hoc analysis with Bonferroni correction. ns, no significant difference; Kruskal–Wallis test followed by post hoc analysis with Bonferroni correction. Con, control group; Sevo, sevoflurane group; Bu + Sevo, Bumetanide + Sevoflurane group; Bu + Con, Bumetanide + Control group

Our study confirmed the results of a previous study (Wei et al., 2019) showing that sevoflurane induces widespread apoptosis in the DG of postnatal day 21 mice. Compared with the Con group, the Sevo group had a significantly higher number of apoptotic dentate granule cells (*n* = 9 for Con group, *n* = 10 for Sevo group, *p* < .001, Kruskal–Wallis test, Figure 1a–f). Quantification of immunofluorescence staining revealed that apoptotic cell death was significantly reduced in the Bu + Sevo group compared with the Sevo group (*n* = 11 for Bu + Sevo group, *p* < .0026, Kruskal–Wallis test), suggesting a protective role for bumetanide in sevoflurane‐induced neuroapoptosis. Furthermore, no statistical difference was found between the Con and Bu + Con groups (*n* = 8 for Bu + Con group, *p* > .05, Kruskal–Wallis test) suggesting that bumetanide does not promote apoptosis in the DG of postnatal day 21 mice. In addition, Qiu et al. (2015) revealed that bicuculline, a GABA_A_ receptor antagonist, could reverse apoptosis in neural stem cells caused by sevoflurane. In our study, we found that compared with Sevo group, the density of apoptotic granule cells was significantly decreased in Bicuculline + Sevo group (*p* = .0055, Kruskal–Wallis test, Figure S1). Compared with bicuculline, bumetanide had similar protective effects on the sevoflurane‐induced neuronal apoptosis in DG (*n* = 11 for per group, *p* > .05, Kruskal–Wallis test, Figure S1). These findings confirmed the protective effect of bumetanide on sevoflurane‐induced apoptosis.

### 3.2 Sevoflurane exposure does not affect the motor ability and anxiety level

**FIGURE 2:**
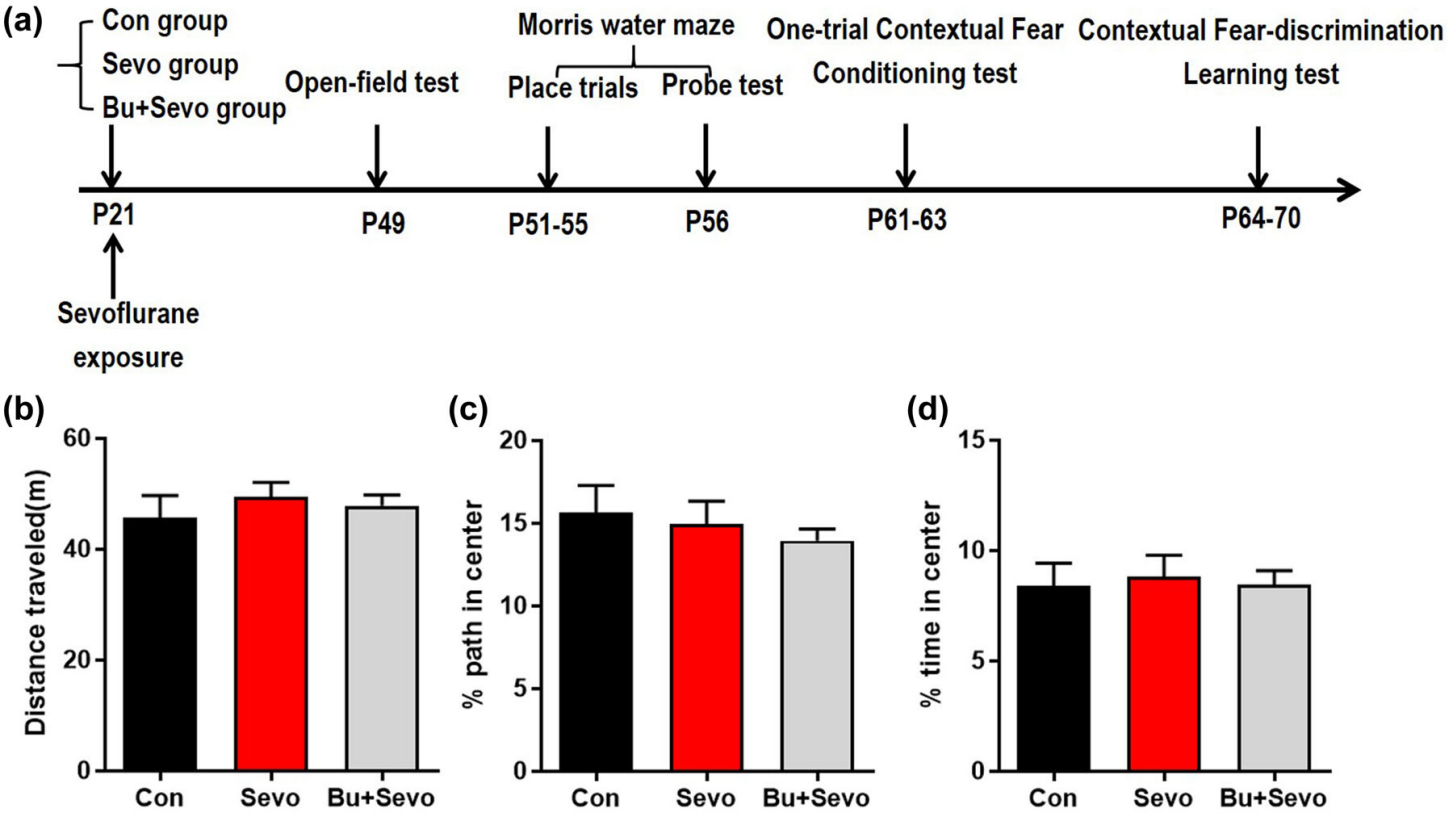
Sevoflurane exposure does not affect the motor ability or anxiety level in mice: (a) flow diagram of the behavioral tests. Open‐field test results for the total path length (b) and the percent of path length (c) and time spent (d) in the center area. Con = control group, Sevo = sevoflurane group, Bu + Sevo = Bumetanide + Sevoflurane group; *n* = 12 for each group

As a previous study indicated that it requires 3–4 weeks for immature dentate granule cells to functionally integrate into the hippocampal circuit, a series of behavioral tests began with an open‐field test 4 weeks after the exposure to anesthesia (Figure 2a). As shown in Figure 2b, there was no significant difference in the total distance traveled among the Con, Sevo, and Bu + Sevo groups (Con: 45.71 ± 3.95, Sevo: 49.47 ± 2.58, Bu + Sevo: 47.83 ± 1.93, data were shown as Mean ± SEM, *n* = 12 for per group, *p* = .65 one‐way ANOVA, Figure 2b). In addition, there was no statistical difference in the distance (Con: 15.65% ± 1.62%, Sevo: 14.95 ± 1.38, Bu + Sevo: 13.93 ± 2.24, data were Mean ± SEM, *p* = .61, one‐way ANOVA, Figure 2c) and time spent in the center area (Con: 8.38 ± 1.04, Sevo: 8.80 ± 2.93, Bu + Sevo: 8.44 ± 2.02, data were mean ± SEM, *p* = .93, one‐way ANOVA, Figure 2d) among the three groups, indicating that postnatal day 21 mice treated with sevoflurane had no obvious changes in their general locomotor activity and anxiety level.

### 3.3 Sevoflurane exposure has no effect on the performance in the MWM

**FIGURE 3:**
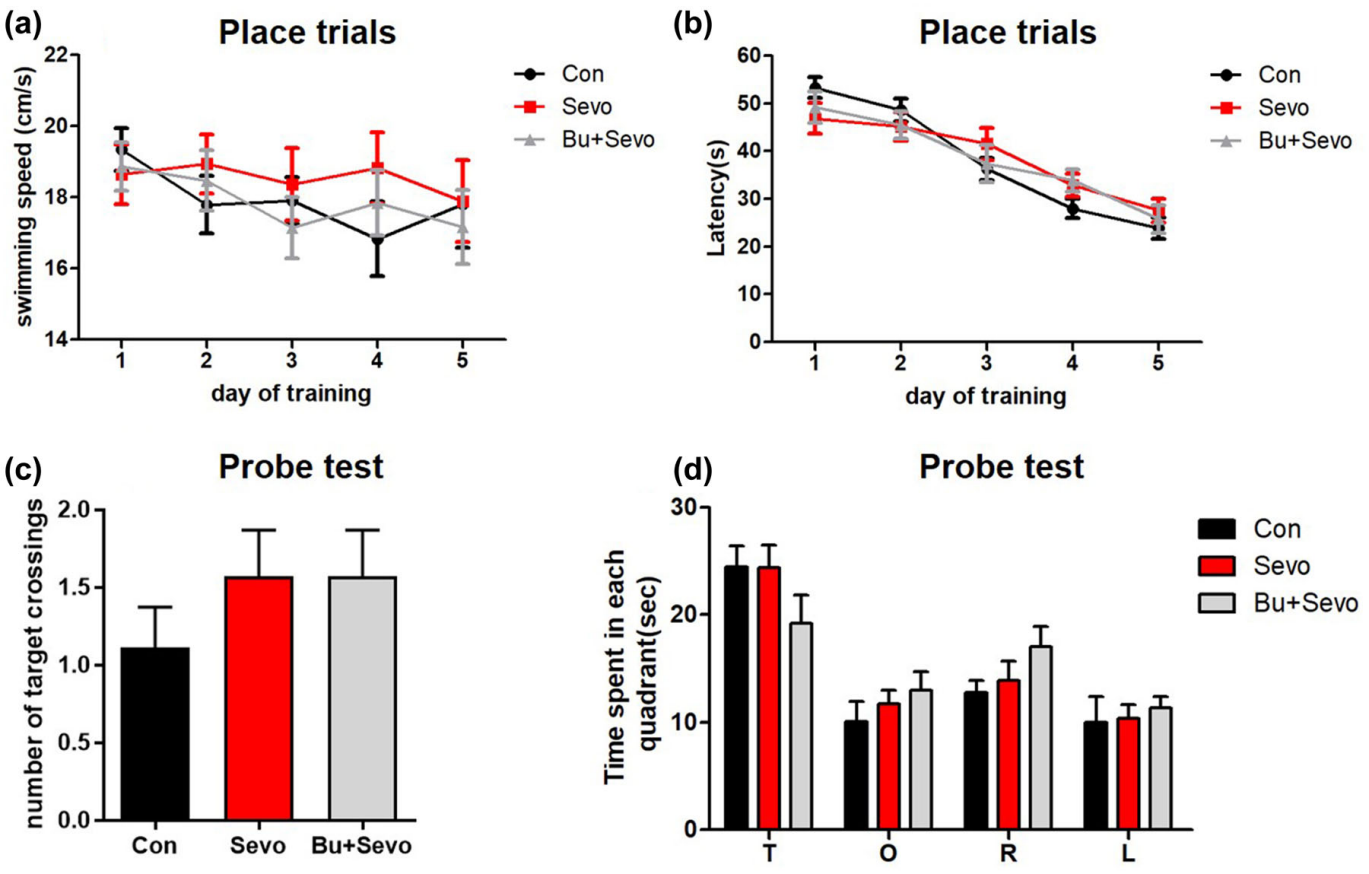
Performance of mice in the Morris water maze (MWM) test: (a) swimming speed in the MWM spatial training task for the Con, Sevo, and Bu + Sevo groups; (b) the latency time in the MWM spatial training task for the same groups; (c) the number of platform crossings during probe trials; (d) time spent in each of four quadrants in the probe trials for the groups. Con = control group, Sevo = sevoflurane group, Bu + Sevo = Bumetanide + Sevoflurane group; *n* = 12 for each group

One day after the open‐field test, mice received the MWM test. The three groups showed no statistical difference in swimming speed (*n* = 12 for per group, *p* > .05 from day 1 to 5 of training, two‐way ANOVA, Figure 3a). No significant difference was observed in the escape latency among the three groups (*p* > .05 from day 1 to 5 of training, two‐way ANOVA, Figure 3b). In addition, there was no significant difference in the number of platform crossings (Con: 1.11 ± 0.26, Sevo: 1.57 ± 0.30, Bu + Sevo: 1.57 ± 0.30, *p* = .26, data were mean ± SEM, Kruskal–Wallis test, Figure 3c) and time spent in the target quadrant among the three groups (*p* > .05 from in T, O, R, L quadrant, one‐way ANOVA, Figure 3d). Collectively, these results suggest that exposure of postnatal day 21 mice to sevoflurane does not affect spatial learning and memory abilities.

### 3.4 Bumetanide alleviates sevoflurane‐induced performance impairment in the contextual fear discrimination learning test

**FIGURE 4:**
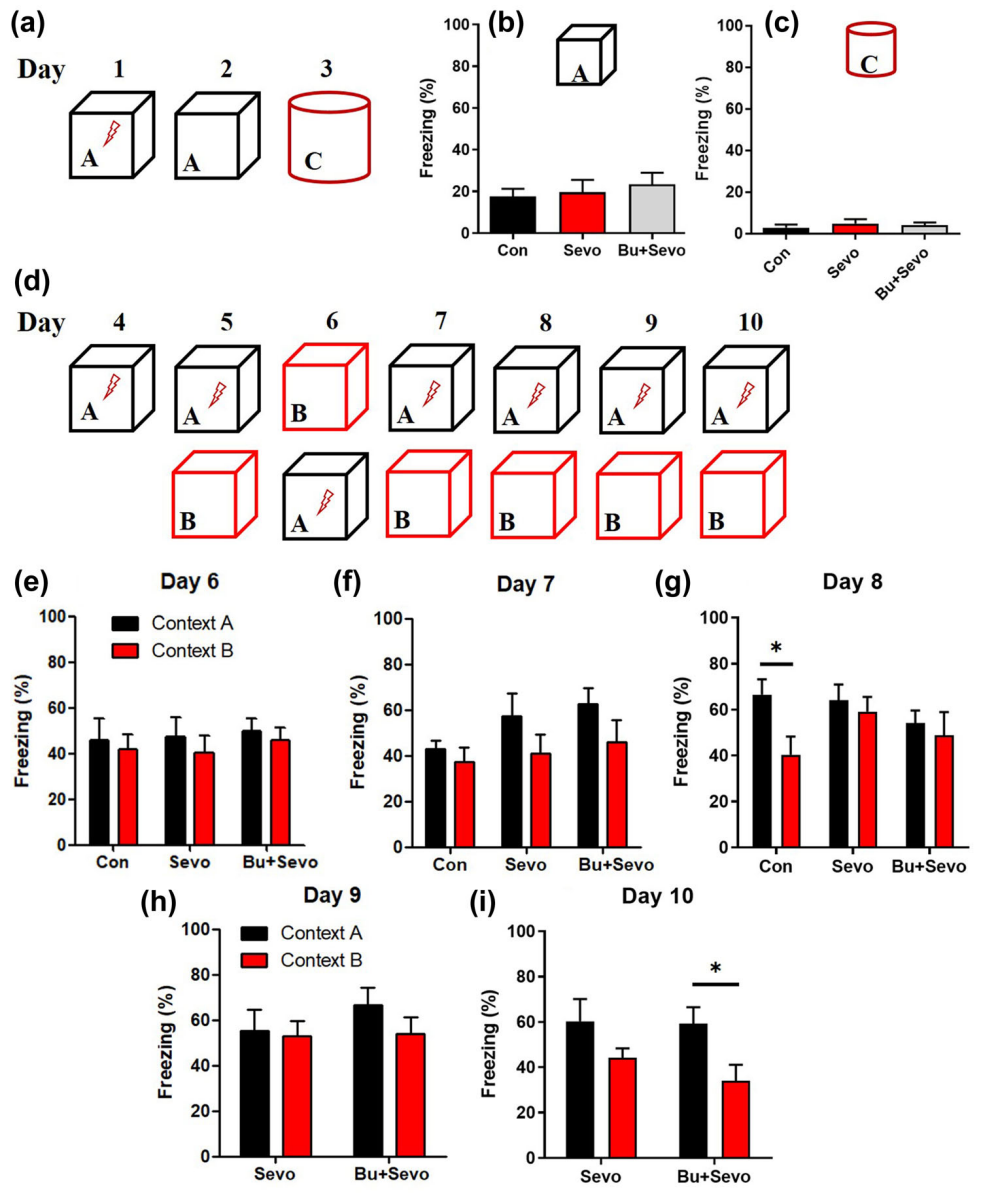
Bumetanide attenuates sevoflurane‐induced impairment in the contextual fear discrimination test: (a) flowchart for the one‐trial contextual fear conditioning test; (b) freezing behavior of each group in the context‐A conditioning context; (c) freezing behavior of each group in the context‐C novel context; (d) experimental flowchart for the contextual fear discrimination learning test; (e)–(i) freezing behavior of each group in the context‐A conditioning context and the context‐B similar context on days 6−10. ^*^ *p* < .05, unpair *t* test. Con = control group, Sevo = sevoflurane, Bu + Sevo = Bumetanide + Sevoflurane group; *n* = 24 for each group. In the Box A, the lights in the box were on, metal floor was exposed, and a mild lemon scent was utilized as an olfactory cue. In the Box B, the walls of chamber were covered with plastic panels, the light was turned off, and the chamber door was ajar during the test. The olfactory cue in context B was a mild pineapple scent, and a nonalcoholic antiseptic was used to clean the chamber between trials. No electronic shock was delivered in the context‐B test. In the Box C, the light was off, two walls of the cage were covered by plastic panels, and the stainless‐steel grid floor was covered with cardboard. No electronic shock was delivered in the context‐C test.

To further explore the potential effects of sevoflurane on behavioral performance, we evaluated contextual fear discrimination learning under conditions that were most likely to recruit the DG. Mice in the Con, Sevo, and Bu + Sevo groups showed similar freezing behaviors in the conditioning context (context‐A) box without electronic shock, after being trained in context A with the shock (Con: 17.51% ± 3.39%, Sevo: 19.63% ± 5.89%, Bu + Sevo: 23.36% ± 5.63%, data were Mean ± SEM, *n* = 24 for per group, *p* = .72, one‐way ANOVA, Figure 4b). When the mice were placed into the novel context (context C) on the third day of the experiment (Figure 4a), the freezing behaviors in the three groups were almost negligible (Con: 2.84% ± 1.57%, Sevo: 4.86% ± 2.12%, Bu + Sevo: 5.15 ± 1.20, data were Mean ± SEM, *p* = .35, Kruskal–Wallis test, Figure 4c). This indicates that the freezing behavior was confined to the specific conditioning context, suggesting that contextual fear memories were acquired in each group.

We next tested the ability of the mice to distinguish between the conditioning context and a similar context (Figure 4d). On day 6, no significant difference was found in the freezing behavior between context A and context B among the three groups (Figure 4e). An analysis of the freezing behavior among the three groups over multiple days showed that mice in the Con group learned to distinguish between context A and context B on day 8 (context A: 66.53% ± 6.67%, context B: 40.38 ± 7.80, data were mean ± SEM, *p* = .021, unpaired t test, Figure 4g), and the Bu + Sevo group was able to discriminate between the two contexts on day 10 (context A: 59.33% ± 7.18%, context B: 34.03 ± 7.10, data were Mean ± SEM, *p* = .023, unpaired t test, Figure 4i). However, the Sevo group was still unable to significantly distinguish between the two contexts at this time (context A: 60.32% ± 9.82%, context B: 44.23 ± 4.14, data were Mean ± SEM, *p* = .14, unpaired t test, Figure 4i). Taken together, these data suggest that sevoflurane exposure at postnatal day 21 impaired the pattern separation ability, and bumetanide attenuated this impairment.

## 4 DISCUSSION

Bumetanide is a loop diuretic and has been used in newborns for decades. Bumetanide works by blocking NKCC1 cotransporter that is highly expressed in immature neurons, thereby altering the chloride gradient and resulting in GABA‐mediated inhibition instead of depolarization. The ability of bumetanide to augment the anti‐seizure effect of phenobarbital was demonstrated in vitro preparations and in a rat model of hypoxic neonatal seizures. In addition, bumetanide was found to augment the neuroprotective effect of phenobarbital in HIE models with hypothermia (El‐Dib & Soul, 2017). In humans, Kahle et al. (2009) reported that bumetanide decreased seizure activity in a human neonate, as shown by continuous electroencephalography monitoring. Our study demonstrated that bumetanide alleviates sevoflurane‐induced granule cell apoptosis in the developing DG of postnatal day 21 mice. We also found that sevoflurane exposure at postnatal day 21 impaired the pattern separation ability in the contextual fear discrimination test, and bumetanide attenuates this impairment. These results indicate a protective role for bumetanide in sevoflurane‐induced neurotoxicity and suggest that it is a promising new drug for protecting against anesthesia‐induced neurotoxicity in the developing brain.

A substantial number of studies indicated GABA‐induced depolarization of neurons can act as a potent trophic factor and is critical for neuronal development. NKCC1‐dependent GABAergic depolarization can derive synaptic network maturation during early hippocampal development (Pfeffer et al., 2009). However, studies also show that GABA‐induced depolarization of neurons is a double‐edged sword and can have a detrimental effect on neurons. Blanquie et al. (2017) demonstrated that GABA‐induced depolarization of neurons was responsible for the apoptosis of Cajal–Retzius neurons in the postnatal mouse neocortex. Moreover, Edwards et al. (2010) demonstrated that GABA_A_‐mediated neuronal depolarization contributes to seizures and neurotoxicity in neonatal mice. Similarly, our study revealed that the NKCC1 inhibitor bumetanide attenuates the sevoflurane‐induced apoptosis of granule cells in the DG of postnatal day 21 mice; this suggests that sevoflurane‐induced apoptosis is caused, at least in part, by potentiating NKCC1‐mediated GABA depolarization of the neuronal membrane.

Because our previous study showed that anesthesia‐induced neuroapoptosis in the DG was delayed in mice, peaking at postnatal day 21 rather than P7 (Hofacer et al., 2013), the mice were exposed to sevoflurane anesthesia at postnatal day 21 in the present study. We chose to expose the mice to sevoflurane at the time of their maximum vulnerability to maximize the effects of DG granule cell apoptosis on long‐term behavior. In many studies on anesthetic‐induced neuroapoptosis and long‐term behavioral disorders in the developing brain, rodents are exposed to anesthesia at P4–P8 (Gill & Pickering, 2021; Li et al., 2019; Lin et al., 2017; Wang et al., 2019; Xu et al., 2019). This is very different from our study. This discrepancy might due to there being differential windows of vulnerability to anesthesia‐induced neurotoxicity among brain regions. We noticed that the vulnerability to anesthesia‐induced neuroapoptosis in the CA1 region of the hippocampus peaked ∼1 week postnatal; the CA1 region is also associated with learning and memory (Deng et al., 2014). This might explain why P4–P8 mice are used more frequently for anesthesia neurotoxicity tests.

Since its invention by the British physiologist Richard G. Morris in 1984, the MWM has been widely used to assess the spatial learning and memory abilities of rodents (Morris, 1984; Vorhees & Williams, 2006). Studies have shown that the MWM performance depends largely on the integrity of the hippocampus structure and function, especially the CA1 region (Ikonomidou et al., 2019; Stackman et al., 2016). Although most studies report that exposure of newborn mice to general anesthetics impairs long‐term spatial learning and memory, a fraction of studies have reported that anesthesia exposure had no effect or even improved the performance in the MWM test. Repeated exposure of newborn rats to 2.6% sevoflurane anesthesia at postnatal day 7, 14, and 21 for 2 h did not change the escape latency, or times of platform crossing in adolescence (P31) or adulthood (P91), in the MWM test (Liang et al., 2017). However, neonatal mice (P4–P6) exposed to 1.8% sevoflurane for 6 h have better performance in the training phase of the MWM test (Chen et al., 2015). Our study revealed that sevoflurane exposure at postnatal day 21 had no effect on the spatial learning or memory ability in the MWM test. The inconsistencies between studies might be due to exposing the animals to different concentrations of anesthesia, the duration of exposure, or the age of the animals at exposure.

It is known that the DG is critical for the hippocampus to successfully discriminate between similar experiences, a process known as pattern separation (Treves et al., 2008). Gilbert et al. reported that when colchicine is used to induce lesions in the DG, animals are unable to distinguish between correct and incorrect food wells. However, this discriminatory ability was not affected by a neurotoxic lesion in the CA1 region; this suggests that the different subfields of the hippocampus are specific to distinct functions, and the DG may be uniquely associated with pattern separation (Gilbert et al., 2001). Previous studies also suggested that different granule cells in the DG perform distinct memory functions. The immature granule cells in the inner half of the granular layer of the DG contribute to pattern separation, whereas the mature dentate granule cells mediate rapid recall by pattern completion (Nakashiba et al., 2012). Our study demonstrated that sevoflurane anesthesia administered at postnatal day 21 induces apoptosis of immature granule cells in the DG and impairs the pattern separation ability later in life; that bumetanide could alleviate this impairment implies that sevoflurane‐induced neuroapoptosis might be linked to the impairment of DG‐dependent learning tasks.

Our study had some limitations. We did not investigate the molecular mechanism underlying the protective effect of bumetanide on sevoflurane‐induced neurotoxicity. Shulga et al. (2012) showed that bumetanide prevented p75^NTR^ upregulation and neuronal death in the lesioned injured neurons in the CA1 and CA3 regions of hippocampus. Although both ours’ and Shulga's study demonstrated the protective effect of bumetanide on neuroapoptosis, the disease model and the brain region were different in these two studies. Whether bumetanide protect sevoflurane‐induced apoptosis of dentate granule cells via upregulation p75^NTR^ needed to be further determined. The evidence that the depolarization of neurons by GABA is required for sevoflurane‐induced neuroapoptosis is indirect. Therefore, further experiments are needed to verify the functional activity of GABA in the DG following exposure to sevoflurane. In addition, we described the effects of bumetanide and sevoflurane exposure on neuroapoptosis and pattern separation ability only in postnatal day 21–age mice. Whether the loss of granule cells at postnatal day 21 extends to cognitive impairment in adolescence or adulthood needs further confirmation.

## 5 CONCLUSION

We found that bumetanide alleviates sevoflurane‐induced apoptosis of dentate granule cells in postnatal day 21 mice and attenuates sevoflurane‐induced impairment in their pattern separation ability. These findings suggest a new perspective into the mechanism of anesthesia‐induced neuroapoptosis and a new direction for reducing the neurotoxicity of anesthesia.

## CONFLICTS OF INTEREST

The authors declare that there is no conflict of interest that could be perceived as prejudicing the impartiality of the research reported.

### PEER REVIEW

The peer review history for this article is available at: https://publons.com/publon/10.1002/brb3.2768.

## Supporting information

Figure S1 Bumetanide had similar protective effect against sevoflurane‐induced apoptosis in DG compared with bicuculline. Apoptotic dentate granule cells were obviously seen in Sevo group after exposure to 3% sevoflurane for 6 h (A–C), but not in Bicuculline + Sevo group (D–F) and Bumetanide + Sevo group (G–I). Scale bars = 100 μm. (J) Box plot showing the density of apoptotic cells in Sevo (*n* = 10), Bicuculline + Sevo group, (*n* = 11), and Bumetanide + Sevo group (*n* = 11) groups. Boxes are 25th–75th percentiles, whiskers are 10th–90th percentiles, and closed circles depict outliers. ^**^ *p* < .01, ^*^ *p* < .1 versus Sevo group, Kruskal–Wallis test followed by post hoc analysis with Bonferroni correction. ns, no significant difference; Kruskal–Wallis test followed by post hoc analysis with Bonferroni correction.

## Data Availability

The data that support the findings of this study are available from the corresponding author upon reasonable request.
