# Supplementary material for: Bumetanide attenuates sevoflurane‐induced neuroapoptosis in the developing dentate gyrus and impaired behavior in the contextual fear discrimination learning test
Source: Brain Behav. 2022 Oct 2;12(11):e2768. doi: 10.1002/brb3.2768 (PMC9660414; doi:10.1002/brb3.2768)
Supplement: Supplementary file 1 — Figure S1 Bumetanide had similar protective effect against sevoflurane‐induced apoptosis in DG compared with bicuculline. Apoptotic dentate granule cells were obviously seen in Sevo group after exposure to 3% sevoflurane for 6 h (A–C), but not in Bicuculline + Sevo group (D–F) and Bumetanide + Sevo group (G–I). Scale bars = 100 μm. (J) Box plot showing the density of apoptotic cells in Sevo (n = 10), Bicuculline + Sevo group, (n = 11), and Bumetanide + Sevo group (n = 11) groups. Boxes are 25th–75th percentiles, whiskers are 10th–90th percentiles, and closed circles depict outliers. ** p < .01, * p < .1 versus Sevo group, Kruskal–Wallis test followed by post hoc analysis with Bonferroni correction. ns, no significant difference; Kruskal–Wallis test followed by post hoc analysis with Bonferroni correction. [file BRB3-12-e2768-s001.docx]

**
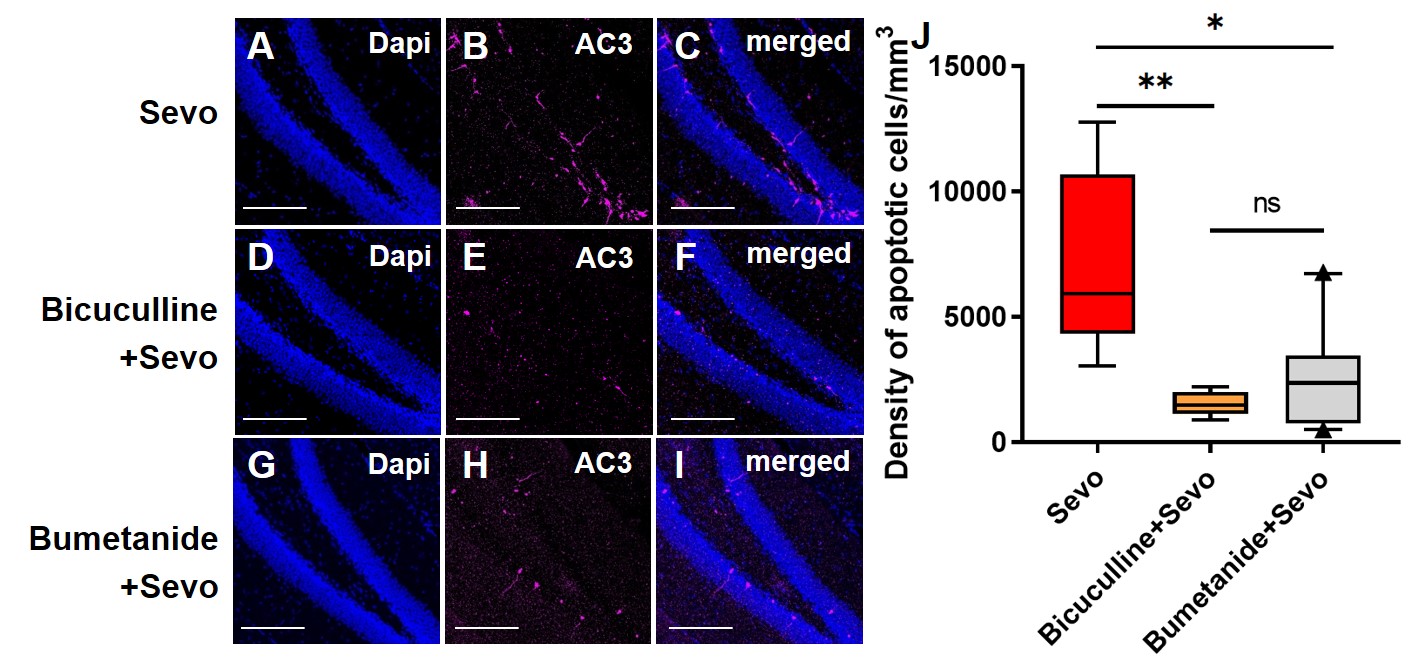
**

**Supplementary Figure 1.** Bumetanide had similar protective effect against sevoflurane-induced apoptosis in DG compared with bicuculline. Apoptotic dentate granule cells were obviously seen in Sevo group after exposure to 3% sevoflurane for 6 h (A-C), but not in Bicuculline+Sevo group (D-F) and Bumetanide+Sevo group (G-I). Scale bars=100 μm. (J) Box plot showing the density of apoptotic cells in Sevo (n=10), Bicuculline+Sevo group, (n=11), and Bumetanide+Sevo group (n=11) groups. Boxes are 25th–75th percentiles, whiskers are 10th–90th percentiles, and closed circles depict outliers. ^**^*P*<0.01, ^*^*P*<0.1 vs Sevo group, Kruskal–Wallis test followed by post hoc analysis with Bonferroni correction. ns: no significant difference, Kruskal–Wallis test followed by post hoc analysis with Bonferroni correction.
